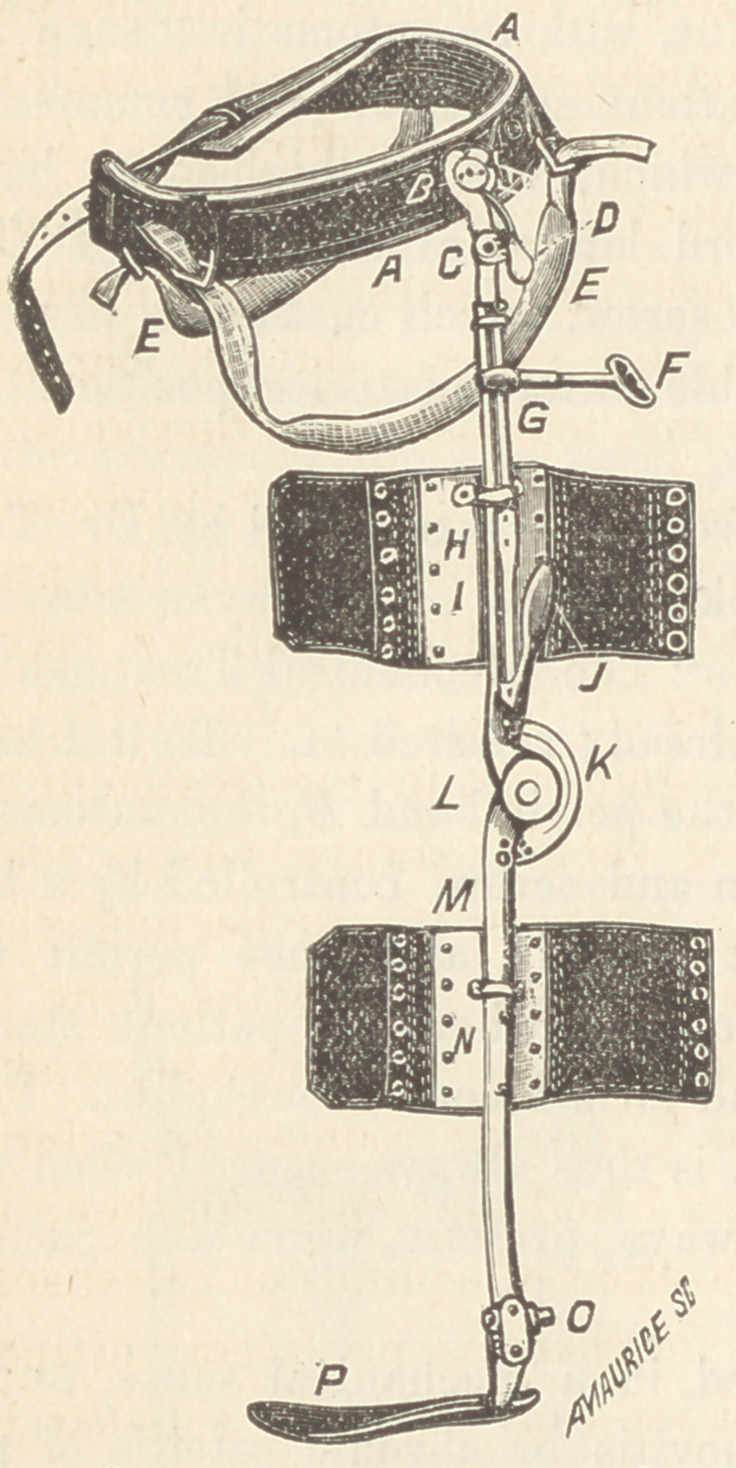# Prognosis and Treatment of Ankle-joint Disease*A lecture delivered at the New York Orthopædic Dispensary and Hospital, November 10, 1881.

**Published:** 1882-06

**Authors:** Newton M. Shaffer

**Affiliations:** New York, Attending Surgeon to the New York Orthapædic Dispensary and Hospital; Orthopædic Surgeon to St. Luke’s Hospital


### THE PROGNOSIS AND TREATMENT OF ANKLE-JOINT DISEASE,*

By NEWTON M. SHAFFER, M. D., of New York, Attending Surgeon to the New York Orthapædic Dispensary and Hospital; Orthopædic Surgeon to St. Luke's Hospital.

In a previous lecture† I called your attention to two important conditions of the ankle-joint—viz.: chronic synovitis and chronic osteitis. Before beginning the consideration of treatment I will briefly call your attention to the prognosis of these lesions.

All joint diseases, and especially those under consideration, require the element of time in their treatment. They are well calculated to test the patience, the industry, the ingenuity, and the temper of the surgeon. In many instances the symptoms will be so unimportant in the minds of the parents, if the patient be a child, or in the consideration of the patient himself, if he be an adult, that complete relief will be looked for in a very short time. But do not let the apparently unimportant symptoms mis-

---

* A lecture delivered at the New York Orthopædic Dispensary and Hospital, November 10, 1881.

† The pathology, symptoms, and diagnosis of Ankle-Joint Disease. *Annals of Anatomy and Surgery*, vol. v., p. 1, January, 1882.

lead you. You may make it a rule to give an unfavorable prognosis in point of time, in certain chronic joint conditions, if the initial symptoms are apparently unimportant. In other words, the nearer the disease approximates an acute inflammation, the better is the prognosis. The more insidious the onset, the longer, as a rule, the disease will continue, and the more uncertain are we regarding the duration, the course, and the ultimate result. Do not attempt, therefore, to give a prognosis until you have duly weighed each symptom, and carefully studied each phase and feature of the history. I have sometimes been tempted to class chronic joint disease as one of the self-limiting lesions. Many of its clinical and pathological aspects lead to the conclusions so ably stated by Dr. Flint regarding phthisis.* In other words, all that we can do in certain cases of chronic joint-disease is to watch, support, and protect just as we do in certain acute diseases; only in many acute lesions the course of the disease is known, and in chronic joint lesions it is not known. Hence, I am very cautious in giving a prognosis in any chronic joint trouble. It is too much like guess-work; and he who guesses in orthopœdic surgery will come to grief. It is my plan to speak frankly to the patient or his friends, and to state plainly both the character of the lesion and the disadvantages under which the surgeon labors. My prognosis, therefore, is rather a description of the nature of the treatment and of the care necessary to obtain a good result. I do not believe that any one is justified in making an exact prognosis in chronic joint disease. I do believe, however, that, unless the case is a very bad one, a good result may be assured in childhood; and this is especially true of ankle-joint disease. But you must appreciate the effort necessary to secure this good result. I cannot speak any more definitely than this. Nor can I speak with any greater certainty until there is some more satisfactory method devised for dealing with the known pathological conditions to which I have called your attention. When we can successfully transform a chronic regressive metamorphosis into an acute reparative lesion we can make a much more favorable prognosis. But we cannot accom-

* See "Self Limitation in Cases f Phthisis," by Austin Flint, M. D. *Archives of Medicine*, vol. I., No. 3.

plish this transformation in all cases; indeed, with our present knowledge, we can succeed in only a very few.

Some surgeons seem to be afraid of producing anchylosis by immobilizing a joint in a state of chronic inflammation. I wish we could accomplish this end by so simple a procedure; but, anchylosis, in chronic arthritis, is not so easily produced. You may stiffen a joint more or less in a state of chronic inflammation, by an immobilizing apparatus, but you will very rarely produce anchylosis by this means. The disease has progressed, and it will progress in certain cases, notwithstanding every effort we may make. It would be a great victory for orthopædic surgery, could some procedure be devised, short of excision of the diseased articular ends, which would produce anchylosis or synostosis when necessary. In the acute joint lesions it is a very different matter. I have seen them rapidly anchylose under immobilization. And I may say that, as a rule, the effort of the surgeon in certain acute joint lesions is to *prevent* anchylosis. On the other hand, in certain forms of chronic articular disease, it is almost impossible to produce this result. In the latter case the condition does not attain a point where repair takes place until, in many instances, the epiphyseal structures are destroyed. The conditions met with are so perplexing and so little understood, and the indications are, apparently, so contradictory in some cases that it is not a matter of surprise that almost every surgeon should deem his own method of treatment the best, when, in reality, all are more or less faulty. We will try to-day to meet the indications as nearly as may be, simply saying that no treatment yet devised meets all the indications that the pathological conditions present.

If we find a distended capsule at the ankle-joint filled with fungous proliferating masses, the first question to be answered is: Does the disease involve the bone? For we should at once recognize the fact that however painless the lesion may be, however slight the inconvenience it occasions, the process is inherently destructive and progressive. Should we decide that the bone is free from disease or only slightly involved, we must at first make every effort to prevent a further progress of the proliferating fungoid masses.

Bear in mind, also, that there are various pathological states in that condition which, for brevity and convenience, I have classed under the general heading of chronic osteitis. Those most frequently met with are:

1. *Osteitis Fungosa or Granulosa*, either primary, *i. e.*, occurring in the epiphyseal structures, or secondary, as a sequence of a fungous degeneration of the synovial tissue.

2. *Caries Necrotica*, a condition in which the process of destruction is rapid, and carious bone is discharged, sometimes in quite large pieces.

3. *Simple Superficial Caries*, following periostitis, and finally:

4. *Caries Interna Caseosa.* The prognosis varies somewhat in all these conditions, as well as the treatment. I have spoken of the prognosis in caries fungosa, and I stop again to impress upon your minds that it is a very sluggish condition, with very insidious prodromata. It is accompanied sometimes by the atonic form of suppuration. You do not get laudable pus in this form of abscess. The discharge is profuse, but thin and ichorous, and filled with broken down tissue. In the necrotic caries the prognosis is more favorable; nature rapidly exfoliates the diseased bone, and the process is more like that which occurs in necrosis of the diaphyses of the long bones. In these cases, too, you will find more marked constitutional disturbance. The process, in other words, more nearly approaches the acute form of disease, and it runs a much shorter course, recovery, with anchylosis, in many instances, taking place. The superficial caries, properly recognized and treated, may also be called a favorable lesion, for it may not involve the articular surfaces. It may, however, become the caries necrotica, and total joint destruction may ensue. Suppuration of the more favorable type frequently accompanies this process, and the rule should be to open as early as possible, contrary to my usual custom. The caries interna caseosa is a form very difficult to recognize, especially in the early stage, and it is very apt, with caries fungosa, to be called tuberculosis of the joint. I do not believe, however, that it is possible to differentiate, clinically, between these two conditions. These two, the fungous or dry caries, and the caseous caries, are forms of disease that will principally engage our attention today. The other forms of disease are those which are more readily recognized and more easily treated, and I will not attempt to discuss them in detail to-day. Nor can I attempt to describe all the chronic joint conditions which we may meet with. Neither can I discuss the various theories regarding their tubercular or non-tubercular origin. I shall rather strive to impress some facts upon your mind regarding the most neglected, and I think the least understood, of these lesions.

But there are those forms of chronic osteitis of the joints to which I have so briefly alluded; and, while in many instances the differential diagnosis is very difficult, especially in the early stage, you may be assured that the treatment to which I am about to call your attention will answer an excellent purpose in all the pathological states mentioned, whether you supplement your mechanical and systemic aids by incision, excision, etc., or merely pursue the method known as the "expectant plan."

There are many points concerning the etiology, pathology, symptoms and prognosis of chronic joint disease, that I should like to call to your attention before proceeding to the consideration of the treatment of ankle-joint disease; but it would be impossible to do the subject justice in one, or even several lectures. We have yet to consider knee, hip and spinal diseases before we close the discussion of these subjects, and you must pardon me if I defer some questions, and more especially those which are particularly suggested by other joint lesions, to a later date.

I showed you, upon the occasion of my previous lecture, two cases of ankle-joint disease. One of them illustrated the clinical aspects of chronic synovitis, the other of chronic osteitis. In the former we found the joint motions nearly normal; a bulging of the joint capsule, an elastic feeling, very suggestive of fluctuation, and an unimpaired walk and attitude. The other presented very different symptoms. The joint motions were very materially impaired by a very pronounced reflex muscular spasm; indeed, anchylosis was simulated by this peculiar condition. The joint outline was not obliterated, and there was no synovial bulging. The patient walked on his heel and limped very markedly; there was also a history of disturbed sleep. A difference in the condition of the muscles was also found in these two cases.

The patients with chronic synovitis presented a well-rounded calf, matching its fellow, while the one with chronic osteitis showed a smaller calf when compared with its mate. The former had neither reflex muscular spasm, nor a localized muscular atrophy, the latter illustrated both of these conditions. I cite these cases again, and once more call your attention to them, because we shall make them the basis of our remarks upon treatment, and shall have made for them apparatus to be applied in your presence during the present course.

But, before we speak of mechanical treatment, let us stop for a moment and discuss constitutional treatment.

There are certain indications which must be plain to all who have studied these cases, but it is very important that we should recognize that we have a regressive lesion—a retrograde rather than a reparative process—to deal with. We will suppose it to be the fungous synovitis or caries, and in all cases, not otherwise specified, we will refer to these conditions in our remarks. What remedy or remedies are there which will act specifically upon this fungoid degeneration. We must answer frankly that that there are none. Our very ignorance upon this subject drives us at once to the ground of all truly conservative surgeons —viz.: that of thorough, systemic treatment. We must make every effort to restore to nature its impaired reparative power; to bring to the local expression of a peculiar and little understood diathesis a greater plastic effort. But here again we are met by a state of affairs that requires much thought and study. Hygiene, exercise, diet, and many other things are to be thought of; but I know of no specific that I can recommend to you as applicable to all cases, or even one class of cases. Each case is a study, from the hereditary tendencies to the local condition of the joint, and it will require much thought, much study, much experience and observation to apply the remedies best adapted to a case of chronic joint disease. But do not neglect constitutional treatment, though you find that your pet remedy does not produce the impression it should; and, on the other hand, do not depend upon local or mechanical treatment alone.

Some cases are so very discouraging that you may almost be forced to admit, as I have done, that but little could be accomplished by constitutional aid, and that the disease was one which would run its course in spite of all treatment, and get well with a certain amount of joint destruction when the period of limitation was reached, or die in the effort. For in some instances, in spite of all you can do, the apparently simple chronic synovitis will progress and become, in addition, a chronic osteitis. Suppuration of the atonic form may follow, and lardaceous degeneration or tubercular meningitis may remove your patient. These very rarely follow in ankle-joint disease; the hip and spine are most likely to be complicated by these conditions. But one should always take them into account. All this may be very discouraging, I know, but we can only learn by looking at things as they are, not by discussing them from a fictitious standpoint. I have seen chronic joint disease recover with remarkable rapidity, so much so that in some cases I have doubted the correctness of my own diagnosis; but I know that we can, in the majority of instances, help nature very much, and by simply removing traumatism and by aiding nature, as indicated, we can secure the must excellent results, and render our patients, while under treatment, active and happy members of society—a condition of affairs not attained by those who ignore the benefits and curative influence of scientifically constructed apparatus.

The secret of successfully treating chronic joint disease, however, lies in its early recognition. Learn, therefore, to recognize these insidious lesions before great damage is done; not only that, teach the mothers and nurses that they may know, that many slight limps attributed to a tight shoe, or to habit, etc., mean something serious. Teach your families that many cases of "worms" and "indigestion" etc., have turned out to be humpback. Tell the mothers of the families you are called upon to attend that a swelled joint may be the commencement of a serious disease; and thus aid in the diffusion of knowledge, which, if properly interpreted, will save more children from deformity and lameness than all the apparatus ever devised for their relief.

The synovial membrane contains absorbents; it has a limited neural distribution, and its vascular supply is good. After the lesion of chronic synovitis has existed for some time, however, its absorbent power is greatly interfered with. In simple hydrarthrosis, where there are unimportant changes in the synovial secretion, the absorbents still act, and one of our greatest hopes in the treatment of chronic synovitis is that the free surface of the membrane is not disintegrated by the fungous masses. Upon this basis, in certain cases, we make use of compression. For this purpose we use a silk, elastic anklet, compressed sponge or a firmly-applied flannel bandage, which latter should be frequently readjusted. We also use such remedies externally as may excite the absorbents, if there be any left, to greater action. Oleate of mercury, iodine, etc., are useful; so are blisters and friction by the hands. A gentle, but persistent surface massage seems to possess the power of assisting in exciting the absorbents. If the joint shows a higher temperature than normal, avoid the use of excitants and apply ice or other cooling applications, continued, if necessary, for a long time. Other remedies, such as the actual cautery, etc., suggest themselves; but they will occur to you as you study your cases, and read, if necessary, the authors whose contributions are so excellent. Study your entire armamentarium of internal remedies, and do not confine yourself for a long time to any one remedy. Iron, the hypophosphites, the various iodides, mercury, etc., etc., are indicated, depending on the actual condition, the hereditary influences and tendencies. I have known mercury and iodide of potassium to accomplish excellent and rapid cures in cases with a syphilitic taint. I have known it to fail in many other cases. Iron is almost always indicated, and a favorite prescription of mine is the plain tinct. ferri chlor., in large doses, combined with an equal quantity of glycerine. It seems in many cases to aid the plastic power more than any other preparation I have used. But I might add a long list of preparations with which you are familiar. I will again state, before commencing the subject of mechanical treatment, that each case is a study in itself, and that you must determine, by experiment, if necessary, the remedy or remedies necessary to aid you in the all-important element of mechanical support.

Whatever value traumatism may have as the cause of chronic joint disease, such as we have been speaking of to-day, there can scarcely be anyone who doubts its relation to the progress of the lesion, when once it is established. It is, at least, my firm opinion, that when a joint or any of its essential structures become diseased, we should adopt means which will, without doubt, remove the element of constant traumatism, produced by locomotion, etc., and which will protect the vulnerable tissues under all circumstances. This support, at the same time, should not materially interfere with exercise and locomotion. How shall we apply this general principle to the conditions we have been studying to-day? Nature rarely presents a pathological condition without, at the same time suggesting, if not a remedy, indications for its use. We learn from the pathological state and a study of the clinical features, how to apply the means of relief. Let me recall what we have said about chronic synovitis, and see what it suggests.

In chronic synovitis there is almost normal use of the joint, with the fatigue after exercise. We should not, therefore, immobilize, for nature does not make the attempt. We should meet the indication, fatigue after exercise, and apply an apparatus which gives absolute joint protection.* In other words, when the patient is sitting, there need be nothing but protection against accidental traumatism; but when the patient walks this protection must be absolute, and the means by which it is to be obtained should act promptly and without the exercise of volition on the part of the patient. These, in brief, are the indications for the mechanical treatment of chronic synovitis of the ankle-joint.

Bear in mind, for it is very important, that you should always arrange your mechanical aids in treatment of chronic joint disease so that it shall always act independently of the patient's will. Make your protecting instrument, practically, part of the patient and part of the affected joint and limb; adjust it so that the patient can not move or step without making the support available; apply it so that whether sitting or standing, running or walking, whether the danger comes from the patient's own indiscretion or from some accidental cause, the joint—the vulner-

* See "The Mechanical Treatment of Synovitis of the Knee-Joint." By C. Fayette Taylor, M. D. *N. Y. Medical Journal*, July, 1873.

able point—is always protected. I have seen so many inefficient methods adopted for these cases, so many truly useless procedures, that I feel that I cannot speak too strongly upon this point. It is true that anything which gives partial support may produce temporary relief; but, when the patient feels this relief, he at once gives the affected joint more liberty, and being not fully, or, so to speak, not automatically protected, a speedy relapse ensues. Do not seem to do your work in orthopædic surgery, but always be sure that you accomplish all that is required when you use a support. Devise or prescribe the instrument yourself, and see that it is so constructed and applied that the principle involved is carried out. Of course, you must rely somewhat upon the discretion of the patient or his nurse; but you can, by study and effort, so construct and so apply your supports that their use may be reduced to the adherence to a few simple rules, and you can soon tell whether or not these rules are obeyed. If they are not, I would candidly advise you to dismiss the patient, and devote your time to some other and more profitable employment.

Mechanical aids are essential to the scientific treatment of chronic joint disease. They will prevent deformity in many cases, if they can do nothing more. In others, they will, where anchylosis is inevitable, permit you to elect the ultimate position of the joint. And you should always have at hand the facilities for adapting the instrument to the indications, and for making the instrument actually do the work required of it. You should master thoroughly the detail; you should always know more than the instrument maker, not necessarily about the manufacture of the apparatus, but about all other points. You should no more go to an instrument maker for advice than you would to a druggist for instruction. You should not send a patient to an instrument maker for treatment, any more than you would recommend a patient for treatment to a pharmacist. You should prescribe your instrument upon a rational basis, just as you would prescribe for an acute disease; and you should be able to tell the instrument maker, if the instrument is faulty, where the fault lies, or, better still, you should be able to correct the fault yourself. Upon this basis I know of no more satisfactory or pleasant work than orthopædic surgery. But, under any other conditions, it is the most unsatisfactory kind of work. The profession must sooner or later appreciate this, and when they do, orthopædic surgery will be properly understood and appreciated, and that kind of talent will be attracted to it which will make its future brilliant. For there is more to be learned, I feel, in orthopædic surgery than in any other branch of surgery; and hence, there is more to be gained by those who diligently follow it, and intelligently study its problems.

To revert more particularly to chronic synovitis again, I said, and I again repeat it, do not immobilize the joint in chronic synovitis. If you do, the joint soon becomes stiff and useless, and the closely observing friends of the patient will inform you—and they will state a fact—that the joint is in a worse condition than before you applied the apparatus. Use of the joint in chronic synovial inflammation, therefore, is indicated. Motion, without pressure, is plainly demanded; in other words, we must avoid traumatic contact of the vulnerable surfaces. To accomplish this we must produce a certain amount of traction, but we must not prevent free movement.

There are no muscular contractions to overcome; there is no deformity of the joint to remove; there is no pain caused by interarticular pressure in chronic synovitis. The idea should be to produce just enough traction upon the joint to transfer the weight of the body to some other point, giving the joint full liberty when the pressure caused by the weight of the body is not present. Clinical experience teaches, in ankle-joint synovitis, that if we apply an apparatus that just prevents the foot from touching the floor when the patient walks, we place the joint in the best local condition for repair.

I may say here that I have been disappointed with all instruments that I have seen, that have been devised for the treatment of ankle-joint disease, whether synovial or osseous, that act upon the ankle-joint alone. In order to perfectly protect the ankle-joint we must limit more or less the movements of the knee-joint. If we wish to immobilize the ankle-joint in locomotion we must also control the knee. This fact we can, in some cases of synovial disease, ignore; but in the vast majority of cases we cannot ignore it; and hence, we must include the knee-joint in our support. But where shall we obtain our counter support? If we are going to actually protect the ankle we must not depend on adhesive plaster and its attachment to the skin for our counter support. This is one of the fallacies that I have tried to combat. Adhesive plaster forms a very excellent and, indeed, the best means of applying direct traction to a distal part of the body, where we have a fixed point to pull against, as in hip disease, or knee disease, or even in ankle disease, if we apply our adhesive plaster to the parts beyond the ankle-joint. We cannot make a reliable fixed point with adhesive plaster. We must go to the perineum for our fixed point, to the tuber ischii, just as we do in hip disease; and we must make our support so that we can control the knee-joint if necessary, and actually support, protect, or apply traction to the ankle-joint as indicated. In chronic synovitis of the ankle-joint, and in chronic osteitis, also in some cases we can allow the patient to bend the knee when he sits down. As I will show you, there is a peculiar arrangement at the hip and knee in the apparatus I use, by which the patient can flex the knee-joint and the hip-joint as he sits down, and this mechanism locks automatically in the straight position when the patient stands erect or walks.

Here, gentlemen, is the instrument I use in ankle-joint disease; it looks formidable when removed from the patient, but when adjusted properly it is one of the most comfortable and efficient I have ever used. It is composed of six essential parts: (1) The pelvic band *A B*, which enables us to make the fixed perineal point of support *E E*.

(2) The automatic hip-joint movement *C*, which admits of flexion when the patient sits down, and which locks without aid when he stands. (3) The thigh part *G*, in which we place the extension rod. To this thigh-piece we attach a plate *H*, for loose lateral attachment to the thigh. (4) The knee-joint, with its automatic "snap" *J*, which always locks when the patient stands up and commences to walk. (5) The calf part *N*, which, like the thigh-piece, has a band and leather lacing to afford lateral attachment. (6) The ankle-joint *O*, with a worm and screw, which enables us to place the foot-piece *P* in any desirable antero-posterior position (see Fig. 1).

This instrument is modified from one introduced by Dr. Taylor, for chronic synovitis of the knee-joint. Dr. Taylor's instrument is pictured in his article on "The Mechanical Treatment of Synovitis of the Knee-Joint," already referred to. To it I have added the extension rod *C*, and the pelvic band *B*, the automatic joint at the hip *C*, and the worm and screw, controlled by a key at the ankle *O*. The joints at the hip and knee permit the patient to sit in an easy position, and when the patient stands they each lock automatically, and form a continuous splint. Protection, with traction, if desired, is thus always secured when the patient sits, and traction is always present when the patient stands.

The instrument is well adapted, in a mechanical sense, to the treatment of either chronic synovitis or chronic osteitis of the ankle-joint. The modifications necessary to meet the indications in these two conditions are made at the ankle-joint. For chronic synovitis we find just how much motion the patient should have, and then, by a "catch" in the sidepiece of the foot-plate (this is not shown in the plates) we limit the flexion and extension of the foot. We then apply the instrument, and generally without any adhesive plaster whatever. The instrument fits the contour of the limb, and we adjust it so that the foot-plate comes closely in contact with the sole of the foot, before the shoe is applied, and with the pelvic band just below the anterior superior spine of the ilium. Then lace the calf piece first; then apply the shoe over the foot-piece. You will be surprised, if you make the instrument fit, how easily the ordinary shoe goes over this foot-piece.

Then lace the shoe quite snugly; then lace the thigh-piece; finally buckle the pelvic band, and secure the perineal pads. Now make gentle traction, with the key just enough to carry the foot-piece, enclosed by the shoe, slightly away from the sole of the foot. Place the patient on his feet. Almost the entire weight of the body falls on the tubera ischii when the affected ankle-joint is used in locomotion. The knee-joint is stiff, or not as you would wish, for, by removing a couple of screws, the automatic spring can be taken off. The ankle is protected against all ordinary traumatic influences, and the apparatus is almost as much a part of the patient as though he had been born with it. Whatever the patient does, the support is constantly acting, and no voluntary effort is needed to protect the inflamed joint surfaces. The risk of traumatism is reduced to a minimum, and you can now satisfactorily commence your systemic treatment. You can always control the amount of motion at the ankle-joint; and at night, if you wish to protect your patient against accidental traumatism, keep the instrument on and apply a bandage in place of the shoe, or adjust some simple form of ankle-joint splint. You ought to insist that the patient should never walk without the support; for if you permit any discretion in the matter, you might as well have no rules at all. If you say that the patient can walk without the support occasionally in the treatment of chronic synovitis, he will sooner or later make it his habit to reverse your rule, and wear the support occasionally. In chronic osteitis, however, there is very little necessity for this rule. The comfort afforded by the apparatus so far exceeds the discomfort occasioned by its use that the patient will much prefer to wear the support.

In using this instrument for chronic osteitis of the ankle, you not only have a different pathological state from that found in chronic synovitis, but you must also make the modification necessary to meet it. You have no malposition of the joint in chronic synovitis, and hence, you can use the apparatus with more or less antero-posterior movement at the ankle. But in chronic osteitis we find reflex muscular spasm, malposition, either antero-posterior or lateral, pain and much greater difficulty in locomotion. Still, exercise in the open air is indicated, and joint protection is much more urgently demanded. How shall we make this instrument meet the indications?

Let me say here for your future guidance, in the treatment of chronic articular osteitis, that you should always adapt your apparatus exactly to the deformity, whatever it may be, and you must, by gradual and very gentle changes, restore the joint to the normal position, or that position, whatever it may be, which you seek. For ankle-joint osteitis I have introduced an antero-posterior worm and screw (*O*, Figs. 1 and 2). We can then, by a key, turn the foot-plate into a position of equinus or calcaneus, and so adapt it to the deformity; for there is no lateral malposition of the ankle-joint itself, and we can bend the side-bar to meet the secondary tarsal malpositions. After we have adapted the foot-piece and the side-bar to the actual deformity, we go through the same process as that just now described—viz.: We lace the calf-piece first, then adjust the shoe, then lace the thigh-piece, and lastly, arrange the pelvic band and perineal pads. We can reënforce this laced shoe with adhesive plaster, if necessary, however, and then make very thorough traction, applying considerable force, and in this way producing that peculiar avoidance of injurious contact which is so grateful to sufferers from chronic osteitis of the joints.

Patients who could not walk before the support is applied will generally walk well after this apparatus is properly adjusted, without any further aid. They do not need crutches or cane. It is only necessary to make the sound limb artificially longer with a high-soled shoe, and advise care in locomotion at first. You do not have to urge these patients to walk; they soon become very active—as active, indeed, as children without joint disease.

Gradually you will change the position of the foot-plate, and the foot will follow it. In a few days you will see the ankle-joint in a good position, and the progress toward recovery fairly commenced. The instrument, as adjusted, but without the shoe or traction, is shown in Fig. 2.

Before giving you some practical demonstrations, I will briefly refer to some other matters: First, regarding abscesses: their presence does not interfere with the use of this support. Bad cases, with œdematous infiltration, sometimes improve very rapidly under its use, and you can very easily arrange the support so that it will not produce any discomfort. Second. If pain be very severe it is advisable to apply adhesive plaster, and to keep the patient in bed, where the proper local treatment can be carried out. Third. Give a few positive rules for the guidance of your patient, and insist upon their being obeyed.

The question of operative interference, excision, etc., is applicable to other joints as well as to the ankle, and I will consider these and other matters upon some subsequent occasion. I may say, however, that I have seen very few cases of ankle-joint disease where I deemed excision or amputation justifiable in a child. —*Annals of Anatomy and Surgery*, *May*, 1882.

## Figures and Tables

**Figure f1:**
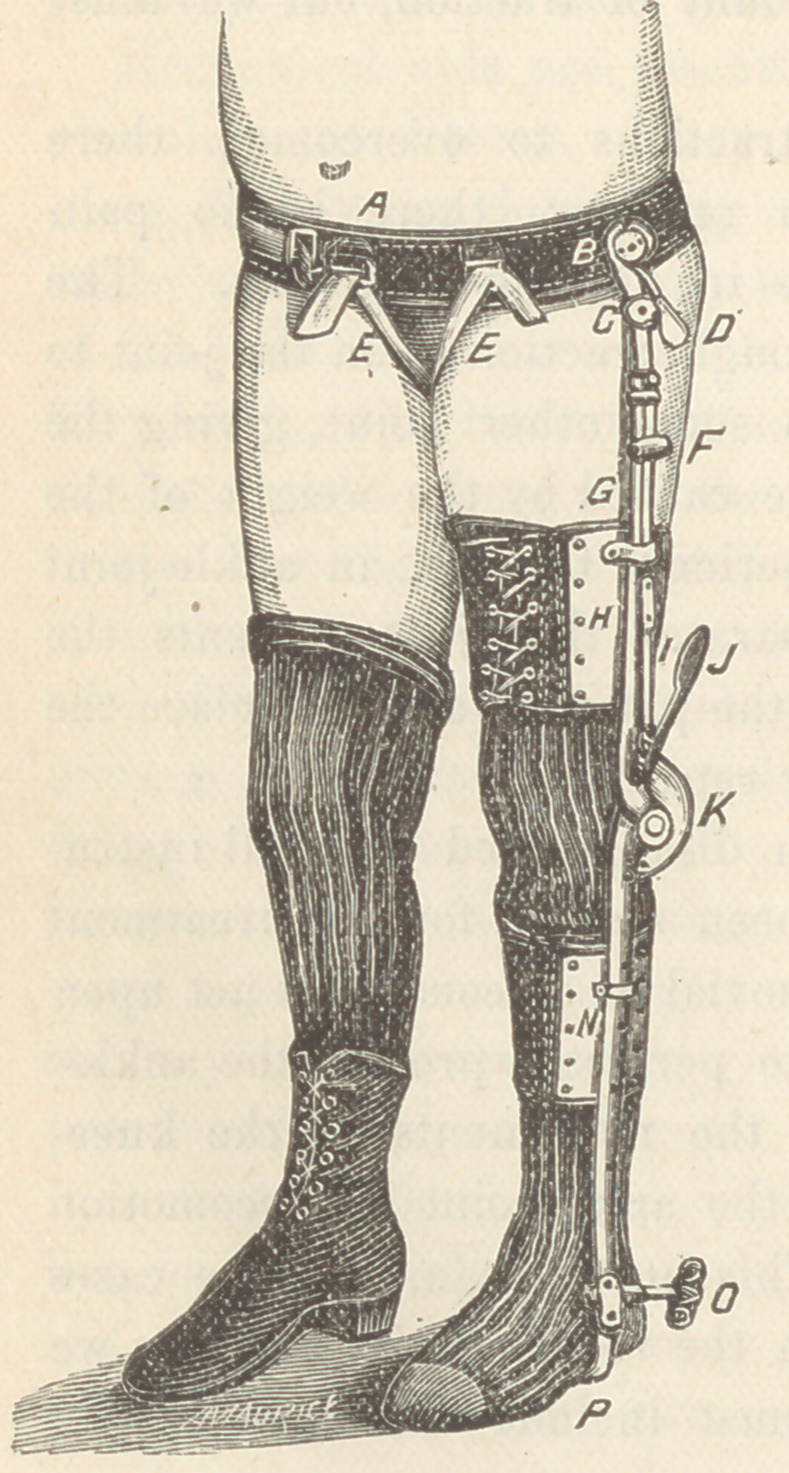


**Figure f2:**